# Supplementary material for: Biologic Drug Survival in Psoriasis: A Systematic Review & Comparative Meta-Analysis
Source: Front Med (Lausanne). 2021 Mar 18;7:625755. doi: 10.3389/fmed.2020.625755 (PMC8012481; doi:10.3389/fmed.2020.625755)
Supplement: Supplementary Figure 1 — Flowchart of study selection process in accordance with PRISMA guidelines. [file Data_Sheet_1.zip › 1. Figure S1.docx]

Studies included in qualitative synthesis
(n= 30)

Full-text articles assessed for eligibility
(n= 36)

Studies included in quantitative synthesis (meta-analysis)
(n= 30)

Records screened
(n= 798)

Additional records identified through other sources
(n= 4)

Records identified through database searching
(n= 1400)

## Identification

Records after 602 duplicates removed
(n= 798)

## Screening

Records excluded (n= 762)

Full-text articles excluded

(n= 6)

## Eligibility

## Included

Figure S1: Flowchart of study selection process in accordance with PRISMA guidelines.
