# Supplementary material for: Biologic Drug Survival in Psoriasis: A Systematic Review & Comparative Meta-Analysis
Source: Front Med (Lausanne). 2021 Mar 18;7:625755. doi: 10.3389/fmed.2020.625755 (PMC8012481; doi:10.3389/fmed.2020.625755)
Supplement: Supplementary Figure 1 — Flowchart of study selection process in accordance with PRISMA guidelines. [file Data_Sheet_1.zip › 2. Table S1.docx]

|  | **Ustekinumab** | | | **Adalimumab** | | | **Etanercept** | | | **Infliximab** | | | **Secukinumab** | |
| --- | --- | --- | --- | --- | --- | --- | --- | --- | --- | --- | --- | --- | --- | --- |
|  | 1 yr. | 2 yr. | 5 yr. | 1 yr. | 2 yr. | 5 yr. | 1 yr. | 2 yr. | 5 yr. | 1 yr. | 2 yr. | 5 yr. | 6mo. | 1yr. |
| **Arnold 2016^1^** | 0.90 | 0.83 | 0.75 | 0.70 | 0.53 | 0.49 | 0.60 | 0.48 | 0.29 | 0.54 | 0.37 | 0.11 | - | - |
| **Davila-Seijo 2016^2^** | 0.80 | 0.62 | 0.39 | 0.67 | 0.45 | 0.22 | 0.56 | 0.38 | 0.17 | 0.66 | 0.47 | 0.26 | - | - |
| **Egeberg 2016^3^** | 0.82 | 0.74 | 0.60 | 0.76 | 0.64 | 0.50 | 0.67 | 0.50 | 0.30 | 0.70 | 0.51 | 0.32 | 0.84 | 0.68 |
| **Esposito 2013^4^** | 0.93 | 0.84 | 0.64 | 0.88 | 0.69 | 0.45 | - | - | - | 0.80 | 0.60 | - | - | - |
| **Gniadecki 2011^5^** | - | - | - | 0.69 | 0.50 | - | 0.74 | 0.58 | - | 0.87 | 0.77 | - | - | - |
| **Gniadecki 2014^6^** | 0.83 | 0.74 | - | 0.75 | 0.65 | 0.50 | 0.70 | 0.55 | 0.34 | 0.72 | 0.61 | 0.42 | - | - |
| **Iskandar 2018^7^** | 0.85 | 0.77 | - | 0.74 | 0.58 | - | 0.49 | 0.36 | - | - | - | - | - | - |
| Izinger 2016 | - | - | - | 0.71 | 0.57 | 0.48 | 0.71 | 0.60 | 0.44 | 0.58 | 0.30 | 0.12 | - | - |
| **Jacobi 2015^8^** | 0.90 | 0.75 | - | 0.70 | 0.54 | 0.44 | 0.74 | 0.49 | 0.24 | 0.51 | 0.17 | - | - | - |
| **Lunder 2018^9^** | 0.80 | 0.72 | - | 0.47 | 0.47 | - | - | - | - | 0.53 | 0.41 | 0.23 | 0.56 | - |
| **Marinas 2018^10^** | 0.91 | 0.86 | 0.76 | 0.80 | 0.65 | 0.51 | 0.83 | 0.69 | 0.42 | 0.84 | 0.66 | 0.38 | - | - |
| **Menter 2016^11^** | 0.95 | 0.90 | - | 0.75 | 0.64 | 0.41 | 0.67 | 0.58 | 0.49 | 0.83 | 0.78 | 0.53 | - | - |
| **Menting 2014^12^** | 0.85 | 0.68 | - | 0.84 | 0.75 | 0.57 | 0.86 | 0.69 | 0.27 | 0.68 | 0.52 | 0.43 | - | - |
| **Ohata 2018^13^** | 0.83 | 0.73 | 0.56 | 0.54 | 0.46 | 0.35 | - | - | - | 0.50 | 0.40 | 0.30 | - | - |
| **Pogacsas 2017^14^** | 0.86 | 0.75 | - | 0.69 | 0.58 | - | 0.72 | 0.60 | - | 0.71 | 0.50 | - | - | - |
| **Ross 2015^15^** | 0.91 | 0.91 | - | 0.73 | 0.53 | - | 0.57 | 0.48 | - | 0.68 | 0.68 | - | - | - |
| **Shalom 2017^16^** | 0.75 | 0.65 | - | 0.29 | 0.21 | - | 0.29 | 0.12 | - | 0.27 |  | - | - | - |
| **Sruamsiri 2018^17^** | 0.8 | 0.73 | 0.52 | 0.46 | 0.46 | - | - | - | - | 0.53 | 0.41 | 0.23 | 0.75 | 0.75 |
| **Verma 2018^18^** | 0.82 | 0.74 | 0.54 | 0.67 | 0.55 | 0.42 | 0.64 | 0.51 | 0.30 | 0.66 | 0.46 | 0.30 | - | - |
| **Vilarrasa 2016^19^** | 0.77 | 0.69 | - | 0.69 | 0.53 | - | 0.71 | 0.53 | - | 0.69 | 0.54 | - | - | - |
| **Warren 2015^20^** | 0.89 | 0.81 | - | 0.79 | 0.67 | - | 0.7 | 0.51 | - | 0.65 | 0.50 | - | - | - |
| **Zweegers 2016^21^** | 0.84 | 0.73 | - | 0.75 | 0.59 | 0.41 | 0.75 | 0.59 | 0.34 | - | - | - | - | - |
| **Cozzani 2019^33^** | 0.99 | 0.81 | 0.62 | 0.77 | 0.61 | 0.32 | 0.80 | 0.62 | 0.35 | 0.75 | 0.57 | 0.28 | 1.00 | 1.00 |
| **Kishimoto 2020^34^** | 0.86 | 0.79 | 0.56 | 0.71 | 0.63 | 0.52 | - | - | - | 0.58 | 0.50 | 0.31 | 0.85 | 0.68 |
| **Shalom 2020^35^** | 0.69 | 0.55 | 0.37 | 0.52 | 0.38 | 0.25 | 0.50 | 0.40 | 0.25 | 0.47 | 0.28 | 0.17 | 0.62 | 0.24 |
| **Egeberg 2018^36^** | 0.82 | 0.74 | 0.60 | 0.76 | 0.64 | 0.49 | 0.68 | 0.50 | 0.30 | 0.71 | 0.52 | 0.32 | 0.84 | 0.67 |
| **Potenza 2017^37^** | - | - | - | 0.84 | 0.74 | 0.65 | 0.81 | 0.72 | 0.53 | 0.71 | 0.51 | 0.36 | - | - |
| **Svedbom 2019^38^** | - | - | - | 0.68 | 0.55 | 0.31 | 0.62 | 0.45 | 0.15 | - | - | - | - | - |
| **Yiu 2020^39^** | 0.90 | 0.83 | - | 0.82 | 0.71 | - | - | - | - | - | - | - | 0.98 | 0.90 |
