# Supplementary material for: Biologic Drug Survival in Psoriasis: A Systematic Review & Comparative Meta-Analysis
Source: Front Med (Lausanne). 2021 Mar 18;7:625755. doi: 10.3389/fmed.2020.625755 (PMC8012481; doi:10.3389/fmed.2020.625755)
Supplement: Supplementary Figure 1 — Flowchart of study selection process in accordance with PRISMA guidelines. [file Data_Sheet_1.zip › Data Sheet 1 (1)/2. Table S1 Supplemental References.docx]

**Supplemental References for Table S1**

1. Arnold TS, Schaarschmidt ML, Herr R, Fischer Je, Goerdt S, Peitsch WK. Drug survival rates and reasons for drug discontinuation in psoriasis. *Journal der Deutschen Dermatologischen Gesellschaft* 2016; **14**: 1089-99.
2. Davila-Seijo P, Dauden E, Carretero G, Ferrandiz C, Vanaclocha F, Gomez-Garcia F *et al.* Biobadaderm Study Group. Survival of classic and biological systemic drugs in psoriasis: results of the BIOBADADERM registry and critical analysis. *Journal of the European Academy of Dermatology & Venereology* 2016; **30**: 1942-50.
3. Egeberg A. Predictors of drug survival for tumour necrosis factor-alpha and interleukin 12/23 antagonists in psoriasis. *British Journal of Dermatology* 2016; **175**: 247-8.
4. Esposito M, Gisondi P, Cassano P, Ferrucci N, Del Giglio M, Loconsole F et al. Survival rate of antitumour necrosis factor-alpha treatments for psoriasis in routine dermatological practice: a multicentre observational study. British Journal of Dermatology 2013; 169: 666-72.
5. Gniadecki R, Kragballe K, Dam TN, Skov TK*.* Comparison of drug survival rates for adalimumab, etanercept and infliximab in patients with psoriasis vulgaris. *The British journal of dermatology* 2011; **164**: 1091-6.
6. Gniadecki R, Bang B, Bryld B, Iversen L, Lasthein S, Skov L*.* Comparison of long-term drug survival and safety of biologic agents in patients with psoriasis vulgaris. *The British journal of dermatology* 2015; **172**: 244-52.
7. Iskandar I, Warren RB, Lunt M, Mason KJ, Evans I, McElhone K et al. Badbir Study Group. Differential Drug Survival of Second-Line Biologic Therapies in Patients with Psoriasis: Observational Cohort Study from the British Association of Dermatologists Biologic Interventions Register (BADBIR). *Journal of Investigative Dermatology* 2018; 138: 775-84.
8. Inzinger M, Wippel-Slupetzky K, Weger, W, Richter L, Mlynek A, Fleischanderl B et al. Survival and effectiveness of tumour necrosis factor-alpha inhibitors in the treatment of plaque psoriasis under daily life conditions: report from the Psoriasis Registry Austria. *Acta dermato-venereologica* 2016,**96**: 207-12.
9. Jacobi AR, Rustenbach SJ, Augustin M. Comorbidity as a predictor for drug survival of biologic therapy in patients with psoriasis. *International journal of dermatology* 2016; **55**: 296-302.
10. Lunder TM, Marko P, Koser Kolar N, Kralj B, Leskovec N. Drug survival of biologic therapies for the treatment of psoriasis: Results of Slovenian national registry. *Biologicals : journal of the International Association of Biological Standardization* 2018; **54**: 44-9.
11. Marinas JEK, Kim WB, Shahbaz A, Qiang JK, Greaves S, Yeung J. Survival rates of biological therapies for psoriasis treatment in real-world clinical practice: A Canadian multicentre retrospective study. *Australasian Journal of Dermatology* 2018; **59**: e11-e4.
12. Menter AP, Papp KA, Gooderham M, Pariser DM, Augustin M, Kerdel FA *et al.* Drug survival of biologic therapy in a large, disease-based registry of patients with psoriasis: results from the Psoriasis Longitudinal Assessment and Registry (PSOLAR). *Journal of the European Academy of Dermatology & Venereology* 2016; **30**: 1148-58.
13. Menting SPS, Sitaram AS, Bonnerjee-van der Stok HM, de Rie MA, Hooft L, Spuls PI*.* Drug survival is not significantly different between biologics in patients with psoriasis vulgaris: a single-centre database analysis. *British Journal of Dermatology* 2014; **171**: 875-83.
14. Ohata C, Ohyama B, Nanri A, Shintani T, Nakama T*.* A retrospective observational study on biological drug treatment in a daily practice serving patients with psoriasis in Japan. *Journal of Dermatological Treatment* 2018: 1-4.
15. Pogacsas LB, Borsi A, Takacs P, Remenyik E, Kemeny L, Karpati S *et al.* Long-term drug survival and predictor analysis of the whole psoriatic patient population on biological therapy in Hungary. *Journal of Dermatological Treatment* 2017; **28**: 635-41.
16. Ross C, Marshman G, Grillo M, Stanford T. Biological therapies for psoriasis: Adherence and outcome analysis from a clinical perspective. *Australasian Journal of Dermatology* 2016; **57**: 137-40.
17. Shalom GC, Cohen A, Ziv M, Eran C, Feldhamer I, Freud T, Berman E *et al.* Biologic drug survival in Israeli psoriasis patients. *Journal of the American Academy of Dermatology* 2017; **76**: 662-9.e1.
18. Sruamsiri R, Iwasaki K, Tang W, Mahlich J. Persistence rates and medical costs of biological therapies for psoriasis treatment in Japan: a real-world data study using a claims database. *BMC dermatology* 2018; **18**: 5.
19. Verma LM, Mayba J, Gooderham JN, Verma A, Papp K*.* Persistency of Biologic Therapies for Plaque Psoriasis in 2 Large Community Practices. *Journal of Cutaneous Medicine & Surgery* 2018; **22**: 38-43.
20. Vilarrasa EN, Notario J, Bordas X, Lopez-Ferrer A, Gich I, Puig L*.* ORBIT (Outcome and Retention Rate of Biologic Treatments for Psoriasis): A retrospective observational study on biologic drug survival in daily practice. *Journal of the American Academy of Dermatology* 2016; **74**: 1066-72.
21. Warren RB, Smith CH, Yiu ZN, Ashcroft DM, Barker JN, Burden DA *et al.* Differential Drug Survival of Biologic Therapies for the Treatment of Psoriasis: A Prospective Observational Cohort Study from the British Association of Dermatologists Biologic Interventions Register (BADBIR). *Journal of Investigative Dermatology* 2015; **135**: 2632-40.
22. Zweegers JZ, van den Reek JM, van de Kerkhof PC, Otero ME, Kuijpers AL, Koetsier MI *et al.* Body mass index predicts discontinuation due to ineffectiveness and female sex predicts discontinuation due to side-effects in patients with psoriasis treated with adalimumab, etanercept or ustekinumab in daily practice: a prospective, comparative, long-term drug-survival study from the BioCAPTURE registry. *British Journal of Dermatology* 2016; **175**: 340-7.
23. Cozzani E, Wei Y, Burlando M*,* Signori A, Parodi A *et al.* Serial biologic therapies in psoriasis patients: A 12-year, single-center, retrospective observational study. *Journal of the American Academy of Dermatology* 2020; **82**: 37-44.
24. Kishimoto M, Komine M, Kamiya K, Sugai J, Mieno M, Ohtsuki M*.* Drug survival of biologic agents for psoriatic patients in a real-world setting in Japan. *The Journal of dermatology* 2020; **47**: 33-40.
25. Shalom G, Cohen AD, Feldhamer I, Comaneshter D, Freud T, Pavlovsky L*.* Drug survival in patients with psoriasis is associated with the availability of biologic medications. *Journal of the European Academy of Dermatology and Venereology : JEADV* 2020.
26. Egeberg A, Bryld LE, Skov L. Drug survival of secukinumab and ixekizumab for moderate-to-severe plaque psoriasis. *Journal of the American Academy of Dermatology* 2019.
27. Potenza MCP, Peris K, Berardesca E, Bianchi L, Richetta A, Bernardini N *et al.* Use of biological drugs in patients with psoriasis and psoriatic arthritis in Italy: Results from the PSONG survey. *Dermatologic therapy* 2018; **31**.
28. Svedbom A, Stahle M. Real-world comparative effectiveness of adalimumab, etanercept and methotrexate: a Swedish register analysis. *Journal of the European Academy of Dermatology and Venereology : JEADV* 2020; **34**: 525-32.
29. Yiu ZZN, Mason KJ, Hampton PJ, Reynolds NJ, Smith CH, Lunt M *et al.* Drug survival of adalimumab, ustekinumab and secukinumab in patients with psoriasis: a prospective cohort study from the British Association of Dermatologists Biologics and Immunomodulators Register (BADBIR). *The British Journal of Dermatology* 2020.
