# Supplementary figures and images for: Biologic Drug Survival in Psoriasis: A Systematic Review & Comparative Meta-Analysis
Source: Front Med (Lausanne). 2021 Mar 18;7:625755. doi: 10.3389/fmed.2020.625755 (PMC8012481; doi:10.3389/fmed.2020.625755)

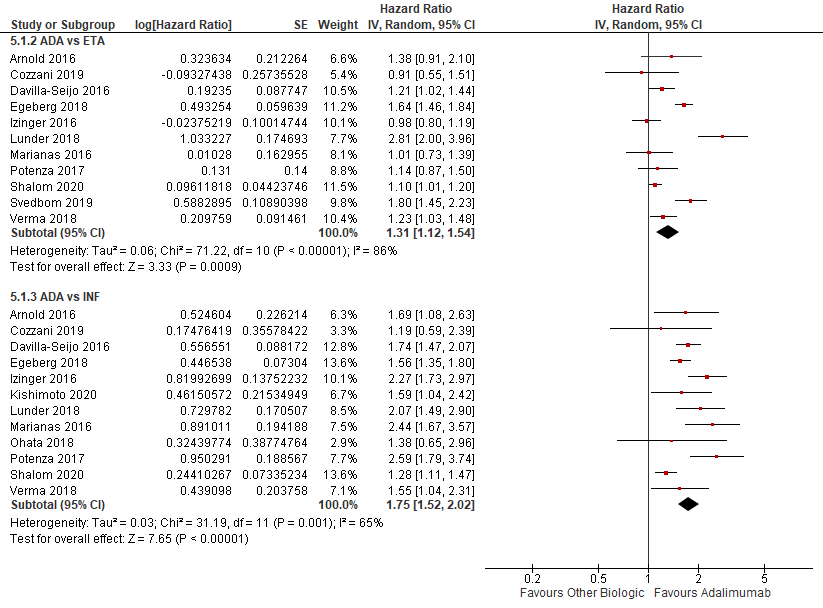

Supplement: Supplementary Figure 1 — Flowchart of study selection process in accordance with PRISMA guidelines. [file Data_Sheet_1.zip › 6. Figure S5.docx]
